# Convergent algorithms for protein structural alignment

**DOI:** 10.1186/1471-2105-8-306

**Published:** 2007-08-22

**Authors:** Leandro Martínez, Roberto Andreani, José Mario Martínez

**Affiliations:** 1 Institute of Chemistry, State University of Campinas, Campinas, SP, Brazil; 2 Department of Applied Mathematics, IMECC-UNICAMP, State University of Campinas, Campinas, SP, Brazil; 3 Department of Applied Mathematics, IMECC-UNICAMP, State University of Campinas, CP 6065, 13081-970, Campinas, SP, Brazil

## Abstract

**Background:**

Many algorithms exist for protein structural alignment, based on internal protein coordinates or on explicit superposition of the structures. These methods are usually successful for detecting structural similarities. However, current practical methods are seldom supported by convergence theories. In particular, although the goal of each algorithm is to maximize some scoring function, there is no practical method that theoretically guarantees score maximization. A practical algorithm with solid convergence properties would be useful for the refinement of protein folding maps, and for the development of new scores designed to be correlated with functional similarity.

**Results:**

In this work, the maximization of scoring functions in protein alignment is interpreted as a Low Order Value Optimization (LOVO) problem. The new interpretation provides a framework for the development of algorithms based on well established methods of continuous optimization. The resulting algorithms are convergent and *increase the scoring functions at every iteration*. The solutions obtained are critical points of the scoring functions. Two algorithms are introduced: One is based on the maximization of the scoring function with Dynamic Programming followed by the continuous maximization of *the same *score, with respect to the protein position, using a smooth Newtonian method. The second algorithm replaces the Dynamic Programming step by a fast procedure for computing the correspondence between C*α *atoms. The algorithms are shown to be very effective for the maximization of the STRUCTAL score.

**Conclusion:**

The interpretation of protein alignment as a LOVO problem provides a new theoretical framework for the development of convergent protein alignment algorithms. These algorithms are shown to be very reliable for the maximization of the STRUCTAL score, and other distance-dependent scores may be optimized with same strategy. The improved score optimization provided by these algorithms provide means for the refinement of protein fold maps and also for the development of scores designed to match biological function. The LOVO strategy may be also used for more general structural superposition problems such as flexible or non-sequential alignments. The package is available on-line at http://www.ime.unicamp.br/~martinez/lovoalign.

## Background

The number of protein structures obtained experimentally becomes larger every year. This large database is the source of data for the study of important problems in structural biology: The classification of protein structures according to their function, and the correlation of sequence and structure. Studies on the classification of proteins available in the Protein Data Bank (PDB) [1] have already provided important insights into the nature of protein evolution and folding [2-4]. With the increase in computer power and the expansion of the database, using this information for protein design and for the characterization of the protein folding landscape (or fold space) is becoming possible [5,6].

Reliable methods for assessing similarities or discrepancies between structures are thus required. Algorithmic reliability is now most important since the description of the fold space is changing from a discrete to a continuous representation of similarities in terms of geometrical measures [6]. It is not clear whether these measures are meaningful if obtained by methods that do not necessarily converge. Furthermore, in protein folding it was recognized that the stable folds are minimizers in complex energy landscapes [7]. If one intends to obtain insights into the landscape of protein folding from similarity measures (scores), these measures must be meaningful. This means that relevant scores must be developed [4,6,8,9], and that the algorithms that perform the alignment must converge to score maximizers (ideally the global maximizers).

Studies on the organization of the protein fold universe employ multidimensional scaling [10-12] or Kernel methods [13]. From scores that measure the similarity between pairs of proteins, distance-like functions are derived, and proteins are represented as points in the 3D-Euclidean space that best fit the distances. The Structure Space Map developed in [11,12] provides good predictions of function similarities in many cases. Pairwise scores, which are the essential ingredients to compute distances, are given by alignment algorithms. The score information may be "crude, noisy, incomplete or inconsistent" [13]. In particular, scores related to very dissimilar proteins are usually badly computed. Obviously, poor scoring information is not an advantage for the mapping project and alignment methods that give reliable similarity measures, even for very different proteins, should be preferred. In this sense, if we define a score as the best possible association between substructures of two proteins, the best way to proceed is to compute the global maximum of the association measures with respect to relative positions. This is the philosophy of the algorithm by Kolodny and Linial [4] which, on the other hand, is unacceptably expensive for the present computer facilities. Therefore, as currently done in Optimization, we must turn to algorithms that *very likely *compute global maximizers and *are guaranteed to *compute critical points (points that satisfy sharp necessary conditions for maximality).

Summing up, algorithms for protein structural comparisons that provide theoretical tools for a more profound analysis of the relationships between sequence, folding, and structure must have some desirable properties: They must converge, in the sense that a solution must always be encountered, and the solutions must have known properties in terms of the similarity function being considered. The algorithm must also be versatile (adaptable for the optimization of diverse merit functions) since the study of the correlations between structural similarity and functionality may require specific similarity measures. Furthermore, a good method must be competitive with current algorithms in terms of computer time.

### Structural alignment algorithms

Algorithms for protein alignment usually fall into two categories: A large group is based on the comparison of relative internal coordinates of the proteins [2,3,14-17] whereas the second class relies on the explicit superposition of the two structures [18-22]. Structural alignment methods are also affected by the measure of similarity being adopted [17]. The more straightforward comparison tool is the Root Mean Square Deviation (RMSD), but RMSD is not a measure of similarity by itself, since it must be accompanied by the number of C*α *atoms of each structure being aligned and is not sensitive to the presence of gaps [23]. Scores that take into account the presence of gaps and automatically incorporate the number of C*α *atoms being compared have been developed [23]. Algorithms for the maximization of such scores or for the minimization of the RMSD under some conditions use different levels of information on the structure of the proteins: From internal distances only, to secondary structures [14,21]. The comparison of the performance of these methods is not trivial, since each one is based on a different score and the packages often do not provide clearly comparable outputs. However, a comprehensive evaluation of several of these methods was recently elaborated and the results obtained suggest that the STRUCTAL algorithm (see below) provides the best alignments to date [19,23]. These results indicate that the search of the maximizer of distance-dependent additive scores is a valuable strategy for obtaining meaningful alignments. All these algorithms are, however, heuristic in the sense that a rigorous characterization of the solutions that they find is not provided, and the relation of such solutions and score maximizers is uncertain.

### Convergent algorithms for protein alignment

Kolodny and Linial [4] introduced a method for solving the Protein Alignment problem whose complexity is polynomial in the number of C*α *atoms. The idea is to use an exhaustive *ε*-grid search in the space of rotations and to exploit the polynomiality of the Dynamic Programming procedure to optimize a score function. They also illustrate their approach by the maximization of the STRUCTAL score, but other scores could be maximized with the same strategy. If one could define a clearly biologically meaningful similarity measure, this algorithm would provide the optimal alignment for any pair of proteins.

The method of Kolodny and Linial obtains the *global *solution (up to a precision *ε *> 0) of the Alignment problem at the expense of an unaffordable computational effort. Therefore, as mentioned in [4], this method is not practical with the present computer facilities, although it sheds light on the complexity of the problem. This is a very common situation in Optimization practice. Methods that converge to global optimizers can be defined but practical methods are based on local optimization principles.

An algorithm is said to be *convergent *if it finds stationary points of the objective function, independently of the initial approximation (this property is called *global convergence *in the Numerical Optimization field [27,28]). We will show that the interpretation of the protein alignment problem in the context of Low Order Value Optimization Theory [29,30] suggests how convergent algorithms can be formulated. Moreover, we will describe how the STRUCTAL algorithm can be modified in order to obtain convergence. Then, we will introduce a new strategy for obtaining the correspondence between the C*α *atoms of the proteins that provide the algorithms with high efficiency while maintaining good score maximization and allows for non-sequential alignments. Numerical experiments will be presented that demonstrate that the methods are also competitive in terms of computer time with state of the art algorithms. Remarks and perspectives are given in the Conclusions. The Methods section contains details of the implementation of the line-search Newtonian algorithm proposed and rigorous convergence proofs.

## Results and Discussion

### General methodological principles

In this section we describe the Low Order Value Optimization problem. We explain the way in which structural alignment can be interpreted as a LOVO problem, and we show how this interpretation naturally suggests robust convergent algorithms for protein alignment.

#### Protein Alignment as a Low Order Value Optimization Problem

Given *f*_1_(*x*), ..., *f*_*m*_(*x*) a finite set of real functions, the Low Order Value Optimization (LOVO) problem consists of finding *x *such that the maximum of *f*_1_(*x*), ..., *f*_*m*_(*x*) is maximal. That is, defining *f*(*x*) = max{*f*_1_(*x*), ..., *f*_*m*_(*x*)} the objective of LOVO is to maximize *f *(*x*). The application of LOVO to more general problems, an equivalent formulation in terms of minimization and suitable definitions of criticality [31,32] are given in [29]. General algorithms of Newtonian type that globally converge to stationary points were defined in [29] and applied to several practical problems. The main ideas of how convergence is obtained with the application of LOVO methods to protein alignment will be given below. Theoretical details and convergence proofs can be found in [29,30].

**Figure 1:**
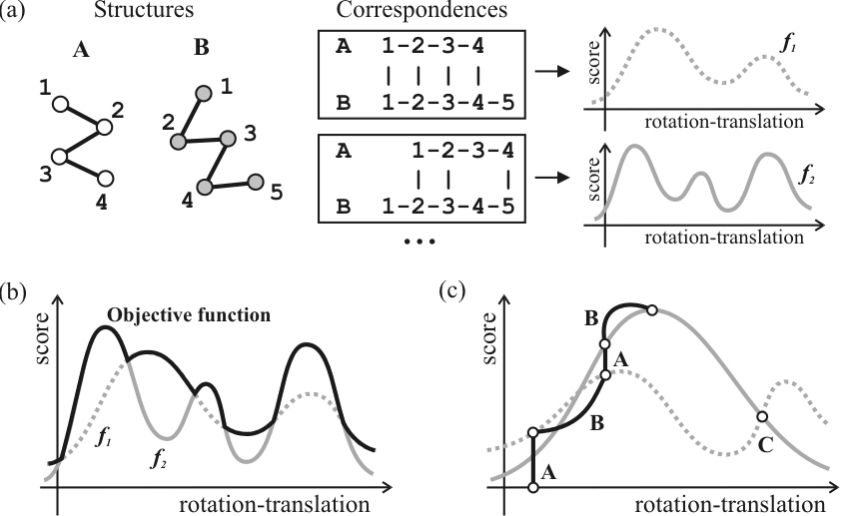
Protein alignment as a Low Order Value Optimization problem. (a) For each correspondence between C*α *atoms of proteins A and B, there is a smooth score that depends on the rotations and translations of the proteins. (b) In protein alignment, the objective function is the function that assumes the maximum value among all the possible score functions. (c) An algorithm that converges to maximizers must have two main steps: A step (A) that recognizes the best correspondence and a step (B) that maximizes the score given the correspondence. Note that at point **C **the objective function is non-smooth.

In Structural Alignment one wishes to obtain the best three-dimensional superposition of two sets of points (atoms). This assumes that there is a correspondence between the atoms of one structure and the atoms of the second structure. Given the correspondence, the structural alignment is usually obtained by the minimization of the RMSD of corresponding C*α *atoms, by rotating and translating one of the structures. Therefore, *each correspondence *defines a function (the RMSD or some other scoring function) of the translations and rotations, as represented schematically in Figure 1(a). Since the number of possible correspondences is finite, the functions of the rigid-body transformations also form a finite set. In theory, one could maximize the scoring function for each correspondence independently, and the correspondence with maximal score value would be the best alignment between the two structures. Therefore, as in the LOVO theory, in structural alignment one wishes to obtain the optimal value of a finite set of real smooth functions (one for each correspondence) [30]. The objective function to be maximized assumes the value of the *f*_*i *_that has the optimal value for each point of the domain, as represented in Figure 1(b).

#### The STRUCTAL algorithm

STRUCTAL [19] was recently reported as being a quite successful algorithm in terms of the quality of the alignments obtained [23]. This indicates that the strategy of maximizing a score that incorporates the distance between atoms, the aligned length and the presence of gaps is valuable for obtaining meaningful alignments. The goal of the STRUCTAL method is to maximize the STRUCTAL score,

STRUCTAL=∑201+(d/2.24)2−10ng
 MathType@MTEF@5@5@+=feaafiart1ev1aaatCvAUfKttLearuWrP9MDH5MBPbIqV92AaeXatLxBI9gBaebbnrfifHhDYfgasaacH8akY=wiFfYdH8Gipec8Eeeu0xXdbba9frFj0=OqFfea0dXdd9vqai=hGuQ8kuc9pgc9s8qqaq=dirpe0xb9q8qiLsFr0=vr0=vr0dc8meaabaqaciaacaGaaeqabaqabeGadaaakeaacqWGtbWucqWGubavcqWGsbGucqWGvbqvcqWGdbWqcqWGubavcqWGbbqqcqWGmbatcqGH9aqpdaaeabqaamaalaaabaGaeGOmaiJaeGimaadabaGaeGymaeJaey4kaSIaeiikaGIaemizaqMaei4la8IaeGOmaiJaeiOla4IaeGOmaiJaeGinaqJaeiykaKYaaWbaaSqabeaacqaIYaGmaaaaaaqabeqaniabggHiLdGccqGHsislcqaIXaqmcqaIWaamcqWGUbGBdaWgaaWcbaGaem4zaCgabeaaaaa@4B2C@

where *d *is the distance between corresponding C*α *atoms and *n*_*g *_is the number of gaps in the sequence alignment.

The apparent success the STRUCTAL method probably comes from the fact that it is based on two strong ideas that are incorporated in several state-of-the-art alignment algorithms: 1) For a given spatial orientation of the two structures, the correspondence that *globally *maximizes a distance-dependent score can be obtained using Dynamic Programming [24]; and 2) For a given correspondence, a rigid-body transformation that *globally *minimizes the RMSD can be obtained analytically (this problem is known as the Procrustes problem, and here we will call "Procrustes" the process of obtaining the rigid-body superposition that minimizes the RMSD between corresponding C*α *atoms of two proteins) [25,26]. The STRUCTAL algorithm consists in the iterative application of these two methods: For the current spatial orientation of the proteins, a correspondence is obtained using Dynamic Programming, and this correspondence is used for a best rigid-body superposition (using Procrustes) in order to obtain a new relative spatial orientation of the structures. The goal of the STRUCTAL algorithm is to maximize the STRUCTAL score.

From the point of view of score maximization given a spatial orientation, the use of Dynamic Programming is quite effective and theoretically justified, since this procedure obtains the bijection that globally maximizes the score itself [24]. However, obtaining the rigid-body transformation that minimizes the RMSD for the current bijection between C*α *atoms is not a score-maximizing strategy. Therefore, although this procedure seems to be adequate because alignments with low RMSD are desirable, it is not the best choice if one wants to maximize the scoring function. In particular, the algorithm usually does not converge to a maximizer of the score, and many times oscillates between two different alignments.

#### Convergent LOVO algorithms for protein alignment

Figures 1(a) and 1(b) show how the objective function of protein alignment can be interpreted as the maximal function of rotations-translations corresponding to each admissible correspondence between C*α *atoms of the two structures.

A convergent method for maximizing this function must have two abilities, as sketched in Figure 1(c):

1. For a given displacement (rotation-translation, giving a three-dimensional relative orientation between the two proteins), a suitable subalgorithm must be able to identify which is the correspondence that maximizes the score. This corresponds to steps of type A in Figure 1(c).

2. Once the best correspondence is found, another subalgorithm must be able to obtain a new rotation-translation displacement that improves the score for the correspondence obtained by the step of Type A. These are the steps of type B in Figure 1(c).

In the context of protein alignment, steps of type A may be performed using the classical Dynamic Programming algorithm [24]. Unfortunately, Procrustes rigid-body superpositions do not satisfy the conditions required for the steps of type B, since they do not guarantee the improvement of the score. However, reliable optimization algorithms exist that are always able to obtain improvements of the score merit function. Therefore, a natural approach consists of replacing Procrustes by an iteration of one of these algorithms. This is the principle governing the methods proposed here. Interestingly, note that point C in Figure 1(c) is non-smooth because two correspondences provide the same score for the given rotation-translation. As justified by the LOVO theory, however, the non-smoothness of the objective function may be simply ignored: A smooth optimization algorithm that takes any of the concurrent gradients may be used for improving the merit function at each iteration [29,30].

## Algorithms

The Low Order Value Optimization approach suggests that, for obtaining a convergent score optimization algorithm, one should replace the Procrustes best rigid-body transformation by some algorithm that guarantees increasing the score during the structural alignment step. Here, we propose a classical Safeguarded Line-Search Newtonian algorithm, leading to the Dynamic-Programming-Line-Search method. Next, motivated by the rather high computational cost of the Dynamic-Programming step, we propose a second LOVO algorithm that preserves the Line-Search step but uses a Non-Bijective correspondence that can be computed very fast. This will be the Non-Bijective-Line-Search method, which will allow also for the study of non-sequential alignments. Both algorithms converge to stationary points of the scores, in the sense defined above.

### Dynamic-Programming-Line-Search method (DP-LS)

The DP-LS method preserves the Dynamic-Programming step. However, it uses a Safeguarded Line Search Newtonian method that guarantees a sufficient increase of the score during the structural alignment step [28].

#### Safeguarded Line Search Newtonian step

**Figure 2:**
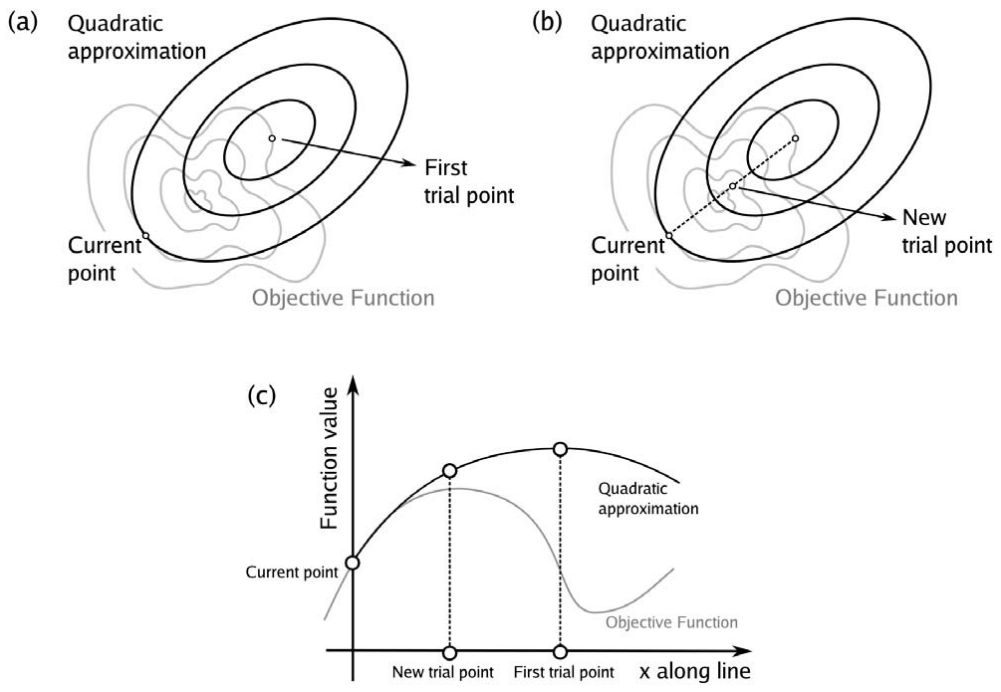
Sketch of the line-search Newtonian method. (a) A quadratic model is obtained and maximized in the first step. (b) If the function value has not increased (enough) a new trail point is obtained closer to the current point. (c) For a trial point close enough to the current point, the objective function must increase.

The basic computations underlying Line Search Newtonian algorithms are the following:

1. Obtain a strictly concave quadratic approximation of the objective function at the current point.

2. Maximize this quadratic approximation. The maximizer of the quadratic model is called (first) trial point, as shown in Figure 2(a).

3. If the true objective function increased enough at the trial point, then the trial point is accepted as the new iterate.

4. If the objective function did not increase enough, a new point is obtained in the segment determined by the current point and the trial point using safeguarded quadratic interpolation. This step is represented in Figure 2(b). In this way one obtains a new trial point and the control returns to Step 3.

The quadratic approximation uses the Hessian matrix, modified in such a way that the model is strictly concave. This guarantees that an ascent direction is generated at every iteration and that the objective function increases sufficiently at every iteration. As a consequence of sufficient increase, every limit point generated by the method is stationary and, under reasonable conditions, the local convergence rate is quadratic.

The reasons why this algorithm converges to a critical point of the objective function are the following: The quadratic model is a good approximation of the true objective function at the current point, since it has the same first and second derivatives than the objective function, as shown in Figure 2(c). Furthermore, the model is concave, so that either the current point is a maximizer of the quadratic model or there is a direction along which the model (and the objective function) must increase. The first trial point is obtained by the maximization of the quadratic model, but it may not increase the function value, as represented in Figure 2(c). If the function value is not increased, a new trial point is obtained, closer to the current point, along the line connecting the current point and the first trial point. Since the parabola that represents the quadratic model along this line is concave, any point in this line must have a greater value for the quadratic model. Furthermore, since the model is a good representation of the true objective function in the vicinity of the current point, for a trial point close enough to the current point, the true objective function must also increase. The strategy of reducing the distance between the current and the trial point guarantees that, eventually, the function value will be improved, and the method must converge to a critical point (see Methods).

#### DP-LS algorithm

Eeach iteration of the DP-LS algorithm is, therefore, defined by the following three steps:

1. Given the three-dimensional orientation, compute the bijection that maximizes the score using Dynamic Programming.

2. Given the current bijection, perform a single Safeguarded Line Search Newtonian iteration to obtain a new orientation for the second protein that guarantees a greater score for the current bijection.

3. If the score increased more than a given tolerance, go to step 1. Otherwise stop.

DP-LS deals with the same objective function at the two phases of the algorithm, in contrast with STRUCTAL, that maximizes the score in the first phase and minimizes the RMSD in the second one. For this reason, we expect that the convergence to score maximizers should be more robust in DP-LS than in the STRUCTAL algorithm. Their computational cost must be similar, since both use the DP step, and the cost of performing a Newtonian iteration is similar to the cost of a Procrustes best rigid-body superposition.

### Non-Bijective-Line-Search method (NB-LS)

Motivated by the fact that Dynamic Programming is computationally expensive, we define here a new correspondence that sacrifices some of the requirements of a good correspondence but that may be computed much more rapidly. Moreover, this procedure makes it possible the generalization of the methods presented here for problems that do not require the correspondence to be monotone and sequential. The NB-LS method can be used for the structural superposition of other structures than proteins, such as ligands.

The Dynamic-Programming step was designed to globally maximize the score for a bijective and monotone correspondence. Both properties are reasonable from the point of view of protein alignment, since the C*α *atoms of each protein are ordered along the protein chain.

We propose now a new (Non-Bijective) correspondence, that sacrifices both bijectivity and monotonicity, but is very cheap to compute: *For each Cα atom of the first protein, the Cα atom corresponding to it is the closest Cα atom of the second protein*. Clearly, this correspondence violates both the bijective and monotone characteristics of the DP correspondence, since different C*α *atoms of the first protein may be associated to the same C*α *atom of the second protein. This correspondence has the somewhat undesirable property that it is not symmetric relative to the interchange of the two proteins. Here we consider systematically the first structure as the smallest one because, as will be shown bellow, this is favorable for obtaining the correspondence rapidly.

This correspondence globally maximizes any score that increases monotonically as the distance between atoms decreases. The STRUCTAL score is one of the many scores that satisfy this property. Therefore, the use of this correspondence associated with the Line-Search Newtonian method also results in a convergent LOVO algorithm, although the functions *f*_*i *_are defined in a different way. The advantage of the non-bijective correspondence is that it may be computed very rapidly, as will be shown below.

#### Fast algorithm for obtaining the NB-correspondence

Our fast algorithm for computing the NB-correspondence involves a preparatory step and an actual algorithmic step. The preparatory step consists in:

1. Obtain the internal distance-matrix of one (usually the largest) protein (called protein B).

2. Sort the elements of each column of the internal distance-matrix. Therefore, the *ordered *internal distance-matrix will contain, for each C*α *atom of protein B, the distances to other C*α *atoms of the same protein in increasing order.

**Figure 3:**
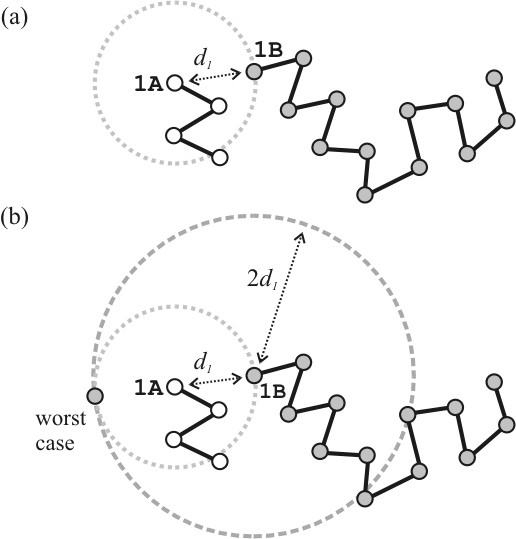
Obtaining the fast non-bijective correspondence: The C*α *atom corresponding to each C*α *atom of protein A is the nearest C*α *atom in protein B. (a) Given the distance *d*_1 _between C*α *atoms 1A and 1B, (b) the C*α *atom of B nearest to 1A is necessarily in the sphere of radius 2*d*_1 _centered in 1B. With an ordered distance matrix for B, only a few distances need to be computed in practice.

Given the ordered internal distance matrix for protein B, one can compute the atom of B being the closest to each atom of A using the following procedure:

1. Compute the distance *d*_1 _of the first atom of A (1A) to some atom of B (for example, to atom 1B), as shown in Figure 3(a).

2. Since we seek the atom of B that is closest to atom 1A, this atom of B cannot be farther than the atom 1B. Therefore, it necessarily belongs to the sphere of radius *d*_1 _around atom 1A, as shown in Figure 3(a).

3. As shown in Figure 3(b), the condition above implies that the atom of B closest to 1A is in the sphere of radius 2*d*_1 _around atom 1B.

4. Therefore, one needs to compute the distances of the C*α *atom 1A *only *to those atoms of B that are closer than 2*d*_1 _to atom 1B. We know which are these atoms because we have the ordered internal distance matrix of B.

In practice, the first distance computed may be to some atom already known to be close to 1A according to previous iterations. For the second atom of A, the first distance computed may be to the atom which was found to be closest to the first atom of A, and so on. These procedures maintain the initial distances *d*_1 _small, keeping also small the number of distances that have to be computed for each atom of A. We observe that the number of distances computed for each atom of the first protein is of the order of 10, almost independently of the size of the protein B.

Using this algorithm for a single protein-protein comparison might not result in time savings due to the cost of the preparatory step. However, when performing database comparisons, the time savings are large because the preparatory steps may be performed in a rational way: For a comparison of a single protein to a database of structures, the ordered internal distance matrix may be computed only for that single protein, which is then systematically treated as protein B. For all-on-all protein comparisons, one computes the ordered distance matrix for the largest protein, treats it as protein B, and aligns it to the whole database. Then we move to the second largest protein, repeat the operation, and so on. Only a single preparatory step is performed for each protein. The computation of the ordered distance matrices was observed to take only about 4% of the total alignment time.

### Testing

The theoretical reasons why the methods presented here should be robust and fast for score maximization were presented in the previous sections. Now we discuss how these methods behave in practice and whether the theoretical expectations were fulfilled.

**Figure 4:**
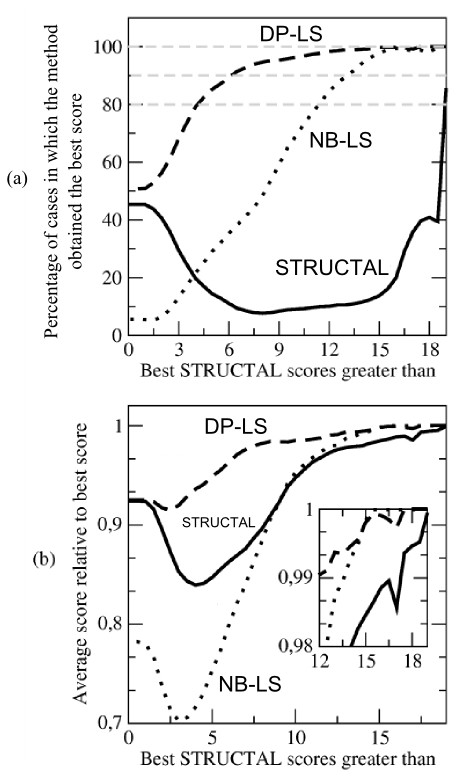
Comparison of the performance of all the methods for STRUCTAL score optimization: (a) Percentage of problems in which each method obtains the best score of all. (b) Relative value of the score obtained by each method relative to the best score obtained by all methods.

Our comparison involves the alignment of 79,800 protein pairs (see the Implementation section for details). For each problem, our first comparison is based on which of the three methods (STRUCTAL, DP-LS or NB-LS) was able to obtain the best score up to relative precision of 10^-3^. Considering all the alignments, the best scores are obtained in about 50% of the cases by the DP-LS method, in 45% of the cases by the STRUCTAL algorithm and in only about 7% of the cases by the NB-LS method. However, most alignments in a database are not meaningful and, therefore, the capacity of the method in identifying good alignments is more important. Therefore, the percentage of best-scores obtained by each method were classified in terms of the best score obtained by the three methods, resulting in Figure 4(a). In this comparison, the STRUCTAL scores are scaled by the number of atoms of the smallest protein involved in each alignment: The STRUCTAL score for a perfect alignment with no gaps is 20*n*_*A*_, where *n*_*A *_is the number of atoms of the smallest protein involved. Therefore, scaling the scores allows one to compare alignments containing proteins with different number of atoms. Scaled scores are always between 0 and 20.

The first clear observation is that DP-LS is systematically able to obtain the best scores in the highest percentage of cases for all alignment qualities. For alignments with (scaled) best-scores greater than 6, for example, DP-LS obtains the best scores in at least 90% of the cases. For alignments with best scores greater than 12, DP-LS obtains the best scores in 98% of the problems. The STRUCTAL algorithm is competitive with DP-LS for bad alignments (scores smaller than 3) and for very good alignments (scores greater than 18).

The NB-LS method obtains the best scores only in about 7% of the cases in general (best-scores greater than zero). However, as the overall quality of the scores increases, the algorithm linearly improves its winning percentage, obtaining the best scores for 90% of the cases for scores greater than 13 and for 98% of the cases for scores greater than 15. In terms of this evaluation, this algorithm obtains better results than STRUCTAL for all scores greater than 4.

Figure 4(a) suggests that DP-LS and NB-LS are very effective for STRUCTAL score maximization. However, this figure does not give a measure of the difference between the scores obtained by each method, providing only a partial image of the actual results. Figure 4(b) is a plot of the average value of the scores obtained by each method relatively to the best scores obtained. Again, the best results are obtained systematically by the DP-LS algorithm, followed by the STRUCTAL algorithm for bad alignments and by the NB-LS algorithm for alignments with best-scores greater than 13.

Figure 4(b) depicts the values of the scores obtained by each method, relatively to the best score obtained. All algorithms obtain scores that are greater than 90% of the best score obtained for problems with best scores greater than 8. For alignments with best scores greater than 14, the STRUCTAL method obtains scores about 1 to 2% smaller than the other methods. This illustrates the effect of using a reliable local convergence strategy on the overall alignment qualities.

These results are as expected in view of the theoretical predictions: The DP-LS algorithm systematically obtains the best results, since it contains only monotone score maximization steps. The best scores obtained by this algorithm are mostly related to the fast local convergence of the LS algorithm. For bad alignments this difference is not as important as it is for good alignments. The NB-LS also behaves as expected: It does not penalize gaps or forces monotonicity during optimization, therefore it is not effective for obtaining good scores for alignments which do not naturally satisfy monotone-bijection and few-gaps properties. However, as the overall alignment quality is improved, these properties are automatically satisfied, and the deleterious effect of the non-bijective correspondence is reduced. For alignments with best scores greater than 13 the sacrifice of the bijection is not as important as the improvement provided by the LS step, and the results are better than the ones obtained by STRUCTAL.

Table 1 shows the average time per alignment in the all-on-all comparisons, obtained for each method. As expected, the NB-LS method is faster than the other two methods, since it replaces the Dynamic-Programming step by a fast algorithm for obtaining the correspondence. It is four times faster than the DP-LS method and six times faster than STRUCTAL. The 79,800 comparisons were performed in 4.6 hours by STRUCTAL, 2.9 hours by DP-LS an in only 44 minutes by the NB-LS method. These relative times were also obtained in a comparison of a single protein to the whole PDB (~34,000 structures). In this case, the CPU time required by the NB-LS method was 19 minutes.

**Table 1:** Average time per alignment of the three methods

Method	Average time per alignment/s
STRUCTAL	0.209
DP-LS	0.133
NB-LS	0.033

**Figure 5:**
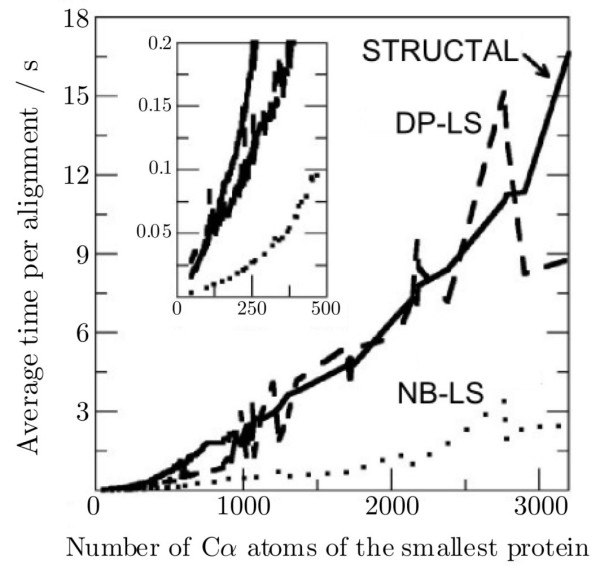
Average time required for the alignment as a function of the size of the smallest protein.

Figure 5 shows the dependence of the alignment time with respect to the size of the proteins being aligned. In Figure 5 we observe that the computational time required for an alignment performed by STRUCTAL or DP-LS increases quadratically as the number of atoms of the smallest protein being compared increases. On the other hand, the complexity of the NB-LS algorithm seems to be almost linear as a function of the number of atoms. Considering only the comparison of the smaller proteins, in the inset of Figure 5, we observe that NB-LS is also quadratic, albeit with a smaller second-order coefficient. The difference in the average time between STRUCTAL and DP-LS is mainly due to the fact that the number of iterations performed by STRUCTAL increases when the size of the proteins being aligned increases (not shown), while the DP-LS obtains convergence in about 10 iterations independently of the size of the proteins being compared. Both the fast local convergence and the non-bijective correspondence provide effective ways to improve the speed of the algorithms. For example, the SSM algorithm [21], which uses secondary structure information, was reported to be about 3 times faster than STRUCTAL, being DP-LS and NB-LS competitive with state of the art algorithms in terms of computer time.

## Implementation

In this section we provide technical details that should be useful to replicate algorithms and experiments.

### Line search

At steps of type B of the algorithms proposed here we used a single line-search procedure. One could perform the full optimization of the score given the current bijection using the LS algorithm until convergence is achieved. However, this is not worthwhile, since after a single movement of the proteins, the bijection that maximizes the score for the new orientation of the proteins frequently changes. Therefore, for every new three-dimensional orientation of the proteins, it is reasonable to recompute the bijection.

Also some safeguards must be taken into account in order to guarantee that the line-search Newtonian method converges. For instance, the current-trial point distances must not be reduced abruptly. Moreover, the increase of objective function value must be at least a fraction of the increase predicted by the quadratic model at the accepted point. The details on how the line-search must be implemented in order to obtain practical and theoretical convergence can be found, for example, in [28] for smooth optimization and in [30] for the (non-smooth) structural alignment problem. More details of the current implementation and convergence proofs can be found in the Methods section.

### Initial approximations

The initial points for the alignments were obtained using an approximate alignment based on the internal coordinates of the proteins: For each protein with *N *atoms, a set of *N *- 3 points in R^3 ^are defined by the distances of atom *i *to atoms *i *+ 2, *i *+ 3, and the distance between *i *+ 2 and *i *+ 3, for *N *consecutive indices *i*. These are the three distances that determine the dihedral of the atoms that follow atom *i*. This creates a "pseudostructure" with *N *- 3 atoms for each protein. The correspondence between the atoms of the pseudostructures of the two proteins is obtained using Dynamic Programming to maximize a STRUCTAL-like score (in which the distances are multiplied by a factor, in our case 20, for providing a reasonable scaling relative to the score parameters). The superposition that minimizes the RMSD for this bijection is obtained using Procrustes and this orientation of the proteins was defined as the initial point for the alignments. This algorithm was observed to directly provide the solution for the alignment of very similar proteins. Furthermore, this method provides good approximations for all algorithms, in such a way that even the classical circularly permuted pair 2pel:A-5cna:A [34] was correctly aligned with all the methods reported here.

For comparing the alignment obtained by the NB-LS method relative to STRUCTAL and NB-LS we compute, as a post-processing step, the actual bijective and monotone STRUCTAL scores relative to the (DP) optimal monotone bijection, for the final alignment obtained.

### Numerical examples

We selected, at random, 20 proteins from the publicly available DALI alignments [3]. For each of these 20 proteins, the 20 best matches found by DALI were also included in our data set. Therefore our database contained 400 proteins, including similar proteins (since they were obtained from a DALI classification) as well as structurally non-correlated proteins. All-on-all alignments within this set of proteins were performed with the three methods, comprising 79,800 alignments for each algorithm. The list of the 400 proteins used can be obtained at the web site of LovoAlign. For sorting the distances during the calculation of the ordered internal distance matrices for the NB-LS method we used the Flashsort algorithm [33]. The methods are implemented in Fortran77. The tests were run on an AMD Opteron 242 with 1 Gb of RAM running Linux. The software was compiled with the GNU fortran compiler version 3.3 with the "-O3 -3ast-math" options.

## Conclusion

Here we presented two contributions, one theoretical, and other practical, to the problem of structural alignment. The theoretical contribution is the interpretation of the alignment problem as a Low Order Value Optimization problem, a framework under which convergent algorithms can be developed. Furthermore, the solutions obtained are critical points of the scoring function. This means that the solutions obtained by our algorithms admit a precise mathematical description in terms of necessary conditions for score maximality.

From the point of view of practical computation, the algorithms presented here seem to be very successful: The study [23] seems to indicate that the STRUCTAL method is a quite robust algorithm for protein alignment. The present paper improves the STRUCTAL algorithm in the following senses:

1. The STRUCTAL algorithm iteration has two phases: Maximizing the STRUCTAL score for fixed positions (Dynamic Programming) and modifying the relative positions with RMSD minimization (Procrustes). These two objectives might be conflictive leading to oscillation. Our DP-LS modifi-cation improves the score at both phases. Therefore, the score increases monotonically at all the iterations.

2. One of the theoretical consequences of the monotone behavior of DP-LS is that this algorithm enjoys convergence to stationary points independently of the initial approximation.

3. The first phase of the STRUCTAL algorithm and DP-LS uses Dynamic Programming. With the aim of reducing the cost of this procedure we introduced a nonbijective correspondence at the first phase of the iterations. The nonbijective association is computationally very cheap.

4. As expected, NB-LS is faster than the algorithms based on first-phase Dynamic Programming. Perhaps surprisingly, for medium to good alignments, its robustness is similar to the one of the STRUCTAL algorithm and DP-LS. The reason is that meaningful alignments usually satisfy the bijective and monotone properties for the best correspondence without being necessary to force them at every step of the optimization procedure.

In this paper, we *do not *address the problem of whether the STRUCTAL score is the best merit function for the evaluation of the biological relevance. The functional relevance of the alignments obtained here is intrinsically linked to the functional relevance of the score being maximized. An ideal score would be one that increases as functional similarity increases. Although several scores have been proposed [8,9,23,34], the design of a score that maximizes biological relevance is still an open problem. This occurs, in part, because reliable practical methods for score optimization were unavailable. The algorithms presented here should be effective tools for the maximization of any distance-dependent score. Therefore, these methods may be used for the development of new scores designed to be functionally meaningful.

The approaches described here can be employed for more general structural alignment problems. The substitution of the Procrustes procedure by the Newtonian algorithm can be used to introduce internal transformations (as flexibility) in the proteins being aligned. On the other hand, the replacement of the Dynamic-Programming strategy by the NB-correspondence makes it possible the treatment of structural alignment problems where monotonicity does not hold. These possibilities were theoretically investigated, and succesfully tested, in a previous work [30], but an effective implementation of these methods including flexibility or other transformation on the structures is an area of future research.

## Methods

Here we give a detailed description of the Newtonian algorithm used in DP-LS and NB-LS and rigorous convergence proofs. More detailed descriptions of the algorithms and of the theory involved can be found in [30]. Although the original problem is given in terms of maximization the maximum of a set of functions, here we use the "minimization of the minimum" approach, which is trivially equivalent. Therefore, our problem may be formulated in the following way.

Minimize *f*_*min*_(*x*)

where

*f*_*min*_(*x*) = min{*f*_1_(*x*), ..., *f*_*m*_(*x*)}.

we denote *I*_*min *_(*x*) = {*i *∈ {1, ..., *m*} | *f*_*i *_(*x*) = *f*_*min*_(*x*)}. we denote ‖z‖=z12+...+zn2
 MathType@MTEF@5@5@+=feaafiart1ev1aaatCvAUfKttLearuWrP9MDH5MBPbIqV92AaeXatLxBI9gBaebbnrfifHhDYfgasaacH8akY=wiFfYdH8Gipec8Eeeu0xXdbba9frFj0=OqFfea0dXdd9vqai=hGuQ8kuc9pgc9s8qqaq=dirpe0xb9q8qiLsFr0=vr0=vr0dc8meaabaqaciaacaGaaeqabaqabeGadaaakeaadaqbdaqaaiabdQha6bGaayzcSlaawQa7aiabg2da9maakaaabaGaemOEaO3aa0baaSqaaiabigdaXaqaaiabikdaYaaakiabgUcaRiabc6caUiabc6caUiabc6caUiabgUcaRiabdQha6naaDaaaleaacqWGUbGBaeaacqaIYaGmaaaabeaaaaa@3E6D@.

**Algorithm A1**. Let *θ *∈ (0, 1), *α *∈ (0, 1/2), *β *> 0, be algorithmic parameters. Let *x*_0 _∈ ℛ
 MathType@MTEF@5@5@+=feaafiart1ev1aaatCvAUfKttLearuWrP9MDH5MBPbIqV92AaeXatLxBI9gBamrtHrhAL1wy0L2yHvtyaeHbnfgDOvwBHrxAJfwnaebbnrfifHhDYfgasaacH8akY=wiFfYdH8Gipec8Eeeu0xXdbba9frFj0=OqFfea0dXdd9vqai=hGuQ8kuc9pgc9s8qqaq=dirpe0xb9q8qiLsFr0=vr0=vr0dc8meaabaqaciaacaGaaeqabaWaaeGaeaaakeaaimaacqWFBeIuaaa@377D@^*n *^be the initial approximation. Given *x*_*k *_∈ ℛ
 MathType@MTEF@5@5@+=feaafiart1ev1aaatCvAUfKttLearuWrP9MDH5MBPbIqV92AaeXatLxBI9gBamrtHrhAL1wy0L2yHvtyaeHbnfgDOvwBHrxAJfwnaebbnrfifHhDYfgasaacH8akY=wiFfYdH8Gipec8Eeeu0xXdbba9frFj0=OqFfea0dXdd9vqai=hGuQ8kuc9pgc9s8qqaq=dirpe0xb9q8qiLsFr0=vr0=vr0dc8meaabaqaciaacaGaaeqabaWaaeGaeaaakeaaimaacqWFBeIuaaa@377D@^*n*^, the steps for computing *x*_*k*+1 _are:

**Step 1. **Choose *ν*(*k*) ∈ *I*_*min*_(*x*_*k*_). If ||∇*f*_*ν*(*k*)_(*x*_*k*_)|| = 0, terminate.

**Step 2. **Compute *d*_*k *_∈ ℛ
 MathType@MTEF@5@5@+=feaafiart1ev1aaatCvAUfKttLearuWrP9MDH5MBPbIqV92AaeXatLxBI9gBamrtHrhAL1wy0L2yHvtyaeHbnfgDOvwBHrxAJfwnaebbnrfifHhDYfgasaacH8akY=wiFfYdH8Gipec8Eeeu0xXdbba9frFj0=OqFfea0dXdd9vqai=hGuQ8kuc9pgc9s8qqaq=dirpe0xb9q8qiLsFr0=vr0=vr0dc8meaabaqaciaacaGaaeqabaWaaeGaeaaakeaaimaacqWFBeIuaaa@377D@^*n *^such that

∇*f*_*ν*(*k*)_(*x*_*k*_)^*T*^*d*_*k *_≤ -*θ*||*d*_*k*_|| ||∇*f*_*ν*(*k*)_(*x*_*k*_)||

and

||*d*_*k*_|| ≥ *β*||∇*f*_*ν*(*k*)_(*x*_*k*_)||.

In the Newtonian version of the algorithm we choose

*d *= - (∇^2^*f*_*ν*(*k*)_(*x*_*k*_) + *λI*)^-1^∇*f*_*ν*(*k*)_(*x*_*k*_),

where *λ *is the first number in the sequence {0,0.1||∇^2^*f*_*ν*(*k*)_(*x*_*k*_)||,0.2||∇^2^*f*_*ν*(*k*)_(*x*_*k*_)||,...} that verifies (2).

The choice (4) corresponds to take *d *= x^
 MathType@MTEF@5@5@+=feaafiart1ev1aaatCvAUfKttLearuWrP9MDH5MBPbIqV92AaeXatLxBI9gBaebbnrfifHhDYfgasaacH8akY=wiFfYdH8Gipec8Eeeu0xXdbba9frFj0=OqFfea0dXdd9vqai=hGuQ8kuc9pgc9s8qqaq=dirpe0xb9q8qiLsFr0=vr0=vr0dc8meaabaqaciaacaGaaeqabaqabeGadaaakeaacuWG4baEgaqcaaaa@2E35@ - *x*_*k *_where x^
 MathType@MTEF@5@5@+=feaafiart1ev1aaatCvAUfKttLearuWrP9MDH5MBPbIqV92AaeXatLxBI9gBaebbnrfifHhDYfgasaacH8akY=wiFfYdH8Gipec8Eeeu0xXdbba9frFj0=OqFfea0dXdd9vqai=hGuQ8kuc9pgc9s8qqaq=dirpe0xb9q8qiLsFr0=vr0=vr0dc8meaabaqaciaacaGaaeqabaqabeGadaaakeaacuWG4baEgaqcaaaa@2E35@ is the minimizer of a quadratic approximation *q*(*x*) of *f*_*ν*(*k*)_(*x*). Namely,

q(x)=f(xk)+∇fν(k)(xk)T(x−xk)+12(x−xk)T(∇2fν(k)(xk)+λI)(x−xk).
 MathType@MTEF@5@5@+=feaafiart1ev1aaatCvAUfKttLearuWrP9MDH5MBPbIqV92AaeXatLxBI9gBaebbnrfifHhDYfgasaacH8akY=wiFfYdH8Gipec8Eeeu0xXdbba9frFj0=OqFfea0dXdd9vqai=hGuQ8kuc9pgc9s8qqaq=dirpe0xb9q8qiLsFr0=vr0=vr0dc8meaabaqaciaacaGaaeqabaqabeGadaaakeaacqWGXbqCcqGGOaakcqWG4baEcqGGPaqkcqGH9aqpcqWGMbGzcqGGOaakcqWG4baEdaWgaaWcbaGaem4AaSgabeaakiabcMcaPiabgUcaRiabgEGirlabdAgaMnaaBaaaleaaiiGacqWF9oGBcqGGOaakcqWGRbWAcqGGPaqkaeqaaOGaeiikaGIaemiEaG3aaSbaaSqaaiabdUgaRbqabaGccqGGPaqkdaahaaWcbeqaaiabdsfaubaakiabcIcaOiabdIha4jabgkHiTiabdIha4naaBaaaleaacqWGRbWAaeqaaOGaeiykaKIaey4kaSYaaSaaaeaacqaIXaqmaeaacqaIYaGmaaGaeiikaGIaemiEaGNaeyOeI0IaemiEaG3aaSbaaSqaaiabdUgaRbqabaGccqGGPaqkdaahaaWcbeqaaiabdsfaubaakiabcIcaOiabgEGirpaaCaaaleqabaGaeGOmaidaaOGaemOzay2aaSbaaSqaaiab=17aUjabcIcaOiabdUgaRjabcMcaPaqabaGccqGGOaakcqWG4baEdaWgaaWcbaGaem4AaSgabeaakiabcMcaPiabgUcaRiab=T7aSjabdMeajjabcMcaPiabcIcaOiabdIha4jabgkHiTiabdIha4naaBaaaleaacqWGRbWAaeqaaOGaeiykaKIaeiOla4caaa@74F6@

If *λ *= 0 (which is the usual case) this is the ordinary Taylor approximation of *f*_*ν*(*k*)_. Sometimes it is necessary to take *λ *> 0 in order to guarantee that a minimum of the quadratic exists and that the generated direction is a descent direction (2). In this case, the geometrical meaning of *d *is that *d *= x^
 MathType@MTEF@5@5@+=feaafiart1ev1aaatCvAUfKttLearuWrP9MDH5MBPbIqV92AaeXatLxBI9gBaebbnrfifHhDYfgasaacH8akY=wiFfYdH8Gipec8Eeeu0xXdbba9frFj0=OqFfea0dXdd9vqai=hGuQ8kuc9pgc9s8qqaq=dirpe0xb9q8qiLsFr0=vr0=vr0dc8meaabaqaciaacaGaaeqabaqabeGadaaakeaacuWG4baEgaqcaaaa@2E35@ - *x*_*k*_, where x^
 MathType@MTEF@5@5@+=feaafiart1ev1aaatCvAUfKttLearuWrP9MDH5MBPbIqV92AaeXatLxBI9gBaebbnrfifHhDYfgasaacH8akY=wiFfYdH8Gipec8Eeeu0xXdbba9frFj0=OqFfea0dXdd9vqai=hGuQ8kuc9pgc9s8qqaq=dirpe0xb9q8qiLsFr0=vr0=vr0dc8meaabaqaciaacaGaaeqabaqabeGadaaakeaacuWG4baEgaqcaaaa@2E35@ minimizes the Taylor quadratic approximation in a restricted trust ball [35].

If *d *satisfies (3) we take *d*_*k *_= *d*. Otherwise, we take *d*_*k *_= *βd*_*k*_/||*d*_*k*_||. In the Newtonian implementation of the algorithm, we use *θ *= 10^-4^, *β *= 10^-6^.

**Step 3**. In this step, we aim to compute *t*_*k *_> 0, *x*_*k*+1 _∈ ℛ
 MathType@MTEF@5@5@+=feaafiart1ev1aaatCvAUfKttLearuWrP9MDH5MBPbIqV92AaeXatLxBI9gBamrtHrhAL1wy0L2yHvtyaeHbnfgDOvwBHrxAJfwnaebbnrfifHhDYfgasaacH8akY=wiFfYdH8Gipec8Eeeu0xXdbba9frFj0=OqFfea0dXdd9vqai=hGuQ8kuc9pgc9s8qqaq=dirpe0xb9q8qiLsFr0=vr0=vr0dc8meaabaqaciaacaGaaeqabaWaaeGaeaaakeaaimaacqWFBeIuaaa@377D@^*n*^, such that

*f*_*min*_(*x*_*k*+1_) ≤ *f*_*min*_(*x*_*k*_) + *αt*_*k*_∇*f*_*ν*(*k*)_(*x*_*k*_)^*T*^*d*_*k*_.

We proceed as follows:

1. *t *← 1;

2. **Suffcient Descent Test**. If *t *satisfies

*f*_*min*_(*x*_*k*+1_) ≤ *f*_*min*_(*x*_*k*_) + *αt*∇*f*_*ν*(*k*)_(*x*_*k*_)^*T*^*d*_*k*_

take *t*_*k *_= *t*, *x*_*k*+1 _= *x*_*k *_+ *t*_*k*_*d*_*k *_and finish Step 3.

3. If *t *does not satisfy (6) compute t^
 MathType@MTEF@5@5@+=feaafiart1ev1aaatCvAUfKttLearuWrP9MDH5MBPbIqV92AaeXatLxBI9gBaebbnrfifHhDYfgasaacH8akY=wiFfYdH8Gipec8Eeeu0xXdbba9frFj0=OqFfea0dXdd9vqai=hGuQ8kuc9pgc9s8qqaq=dirpe0xb9q8qiLsFr0=vr0=vr0dc8meaabaqaciaacaGaaeqabaqabeGadaaakeaacuWG0baDgaqcaaaa@2E2D@ as:

t^=−∇fν(k)(xk)Tdkt22(fmin(xk+tdk)−fmin(xk)−∇fν(k)(xk)Tdkt.
 MathType@MTEF@5@5@+=feaafiart1ev1aaatCvAUfKttLearuWrP9MDH5MBPbIqV92AaeXatLxBI9gBaebbnrfifHhDYfgasaacH8akY=wiFfYdH8Gipec8Eeeu0xXdbba9frFj0=OqFfea0dXdd9vqai=hGuQ8kuc9pgc9s8qqaq=dirpe0xb9q8qiLsFr0=vr0=vr0dc8meaabaqaciaacaGaaeqabaqabeGadaaakeaacuWG0baDgaqcaiabg2da9maalaaabaGaeyOeI0Iaey4bIeTaemOzay2aaSbaaSqaaGGaciab=17aUjabcIcaOiabdUgaRjabcMcaPaqabaGccqGGOaakcqWG4baEdaWgaaWcbaGaem4AaSgabeaakiabcMcaPmaaCaaaleqabaGaemivaqfaaOGaemizaq2aaSbaaSqaaiabdUgaRbqabaGccqWG0baDdaahaaWcbeqaaiabikdaYaaaaOqaaiabikdaYiabcIcaOiabdAgaMnaaBaaaleaaieGacqGFTbqBcqGFPbqAcqGFUbGBaeqaaOGaeiikaGIaemiEaG3aaSbaaSqaaiabdUgaRbqabaGccqGHRaWkcqWG0baDcqWGKbazdaWgaaWcbaGaem4AaSgabeaakiabcMcaPiabgkHiTiabdAgaMnaaBaaaleaacqGFTbqBcqGFPbqAcqGFUbGBaeqaaOGaeiikaGIaemiEaG3aaSbaaSqaaiabdUgaRbqabaGccqGGPaqkcqGHsislcqGHhis0cqWGMbGzdaWgaaWcbaGae8xVd4MaeiikaGIaem4AaSMaeiykaKcabeaakiabcIcaOiabdIha4naaBaaaleaacqWGRbWAaeqaaOGaeiykaKYaaWbaaSqabeaacqWGubavaaGccqWGKbazdaWgaaWcbaGaem4AaSgabeaakiabdsha0baacqGGUaGlaaa@7497@

(If the denominator of the expression above vanishes, we take t^
 MathType@MTEF@5@5@+=feaafiart1ev1aaatCvAUfKttLearuWrP9MDH5MBPbIqV92AaeXatLxBI9gBaebbnrfifHhDYfgasaacH8akY=wiFfYdH8Gipec8Eeeu0xXdbba9frFj0=OqFfea0dXdd9vqai=hGuQ8kuc9pgc9s8qqaq=dirpe0xb9q8qiLsFr0=vr0=vr0dc8meaabaqaciaacaGaaeqabaqabeGadaaakeaacuWG0baDgaqcaaaa@2E2D@ = 0.5.)

With the choice (7), t^
 MathType@MTEF@5@5@+=feaafiart1ev1aaatCvAUfKttLearuWrP9MDH5MBPbIqV92AaeXatLxBI9gBaebbnrfifHhDYfgasaacH8akY=wiFfYdH8Gipec8Eeeu0xXdbba9frFj0=OqFfea0dXdd9vqai=hGuQ8kuc9pgc9s8qqaq=dirpe0xb9q8qiLsFr0=vr0=vr0dc8meaabaqaciaacaGaaeqabaqabeGadaaakeaacuWG0baDgaqcaaaa@2E2D@ is the minimizer of the one-dimensional quadratic (parabola) *φ *that interpolates *f*_*min *_at *x*_*k *_and *x*_*k *_+ *td*_*k *_along the direction *d*_*k*_. By this we mean that

*φ*(0) = *f*_*min*_(*x*_*k*_), *φ'*(0) = ∇*f*_*ν*(*k*)_(*x*_*k*_)^*T*^*d*_*k*_, *φ*(*t*) = *f*_*min*_(*x*_*k *_+ *td*_*k*_).

If t^
 MathType@MTEF@5@5@+=feaafiart1ev1aaatCvAUfKttLearuWrP9MDH5MBPbIqV92AaeXatLxBI9gBaebbnrfifHhDYfgasaacH8akY=wiFfYdH8Gipec8Eeeu0xXdbba9frFj0=OqFfea0dXdd9vqai=hGuQ8kuc9pgc9s8qqaq=dirpe0xb9q8qiLsFr0=vr0=vr0dc8meaabaqaciaacaGaaeqabaqabeGadaaakeaacuWG0baDgaqcaaaa@2E2D@ > *t*/2 we take *t *← *t*/2. If t^
 MathType@MTEF@5@5@+=feaafiart1ev1aaatCvAUfKttLearuWrP9MDH5MBPbIqV92AaeXatLxBI9gBaebbnrfifHhDYfgasaacH8akY=wiFfYdH8Gipec8Eeeu0xXdbba9frFj0=OqFfea0dXdd9vqai=hGuQ8kuc9pgc9s8qqaq=dirpe0xb9q8qiLsFr0=vr0=vr0dc8meaabaqaciaacaGaaeqabaqabeGadaaakeaacuWG0baDgaqcaaaa@2E2D@ <*t*/10 we take *t *← *t*/10. Otherwise, take *t *← t^
 MathType@MTEF@5@5@+=feaafiart1ev1aaatCvAUfKttLearuWrP9MDH5MBPbIqV92AaeXatLxBI9gBaebbnrfifHhDYfgasaacH8akY=wiFfYdH8Gipec8Eeeu0xXdbba9frFj0=OqFfea0dXdd9vqai=hGuQ8kuc9pgc9s8qqaq=dirpe0xb9q8qiLsFr0=vr0=vr0dc8meaabaqaciaacaGaaeqabaqabeGadaaakeaacuWG0baDgaqcaaaa@2E2D@. (This procedure is known as *safeguarded quadratic interpolation *[27].) Go to **Suffcient Descent Test**.

We say that x_* _is a critical (or stationary) point if ∇*f*_*i*_(*x*) = 0 for some *i *∈ *I*_*min*_(*x*). Critical points are Clarke Stationary points in the sense used in [31], for example.

In the following theorems we prove that the algorithm stops at *x*_*k *_only if *x*_*k *_is critical and that limit points of sequences generated by Algorithm **A1 **are critical. These proofs are adaptations of the ones displayed in [29] in a more general setting.

**Theorem 1**. *Algorithm ***A1 ***is well-defined and terminates at x*_*k *_*only if x*_*k *_*is critical*.

*Proof*. Assume that *x*_*k *_is not critical and define *i *= *ν*(*k*). So, ∇*f*_*i*_(*x*_*k*_) ≠ 0. By (2) and the differentiability of *f*_*i*_,

lim⁡t→0fi(xk+tdk)−fi(xk)t=∇fi(xk)Tdk<0.
 MathType@MTEF@5@5@+=feaafiart1ev1aaatCvAUfKttLearuWrP9MDH5MBPbIqV92AaeXatLxBI9gBaebbnrfifHhDYfgasaacH8akY=wiFfYdH8Gipec8Eeeu0xXdbba9frFj0=OqFfea0dXdd9vqai=hGuQ8kuc9pgc9s8qqaq=dirpe0xb9q8qiLsFr0=vr0=vr0dc8meaabaqaciaacaGaaeqabaqabeGadaaakeaadaWfqaqaaiGbcYgaSjabcMgaPjabc2gaTbWcbaGaemiDaqNaeyOKH4QaeGimaadabeaakmaalaaabaGaemOzay2aaSbaaSqaaiabdMgaPbqabaGccqGGOaakcqWG4baEdaWgaaWcbaGaem4AaSgabeaakiabgUcaRiabdsha0jabdsgaKnaaBaaaleaacqWGRbWAaeqaaOGaeiykaKIaeyOeI0IaemOzay2aaSbaaSqaaiabdMgaPbqabaGccqGGOaakcqWG4baEdaWgaaWcbaGaem4AaSgabeaakiabcMcaPaqaaiabdsha0baacqGH9aqpcqGHhis0cqWGMbGzdaWgaaWcbaGaemyAaKgabeaakiabcIcaOiabdIha4naaBaaaleaacqWGRbWAaeqaaOGaeiykaKYaaWbaaSqabeaacqWGubavaaGccqWGKbazdaWgaaWcbaGaem4AaSgabeaakiabgYda8iabicdaWiabc6caUaaa@5DA2@

Then,

lim⁡t→0fi(xk+tdk)−fi(xk)t∇fi(xk)Tdk=1.
 MathType@MTEF@5@5@+=feaafiart1ev1aaatCvAUfKttLearuWrP9MDH5MBPbIqV92AaeXatLxBI9gBaebbnrfifHhDYfgasaacH8akY=wiFfYdH8Gipec8Eeeu0xXdbba9frFj0=OqFfea0dXdd9vqai=hGuQ8kuc9pgc9s8qqaq=dirpe0xb9q8qiLsFr0=vr0=vr0dc8meaabaqaciaacaGaaeqabaqabeGadaaakeaadaWfqaqaaiGbcYgaSjabcMgaPjabc2gaTbWcbaGaemiDaqNaeyOKH4QaeGimaadabeaakmaalaaabaGaemOzay2aaSbaaSqaaiabdMgaPbqabaGccqGGOaakcqWG4baEdaWgaaWcbaGaem4AaSgabeaakiabgUcaRiabdsha0jabdsgaKnaaBaaaleaacqWGRbWAaeqaaOGaeiykaKIaeyOeI0IaemOzay2aaSbaaSqaaiabdMgaPbqabaGccqGGOaakcqWG4baEdaWgaaWcbaGaem4AaSgabeaakiabcMcaPaqaaiabdsha0jabgEGirlabdAgaMnaaBaaaleaacqWGPbqAaeqaaOGaeiikaGIaemiEaG3aaSbaaSqaaiabdUgaRbqabaGccqGGPaqkdaahaaWcbeqaaiabdsfaubaakiabdsgaKnaaBaaaleaacqWGRbWAaeqaaaaakiabg2da9iabigdaXiabc6caUaaa@5CA0@

Since *α *< 1, for *t *small enough we have:

fi(xk+tdk)−fi(xk)t∇fi(xk)Tdk≥α.
 MathType@MTEF@5@5@+=feaafiart1ev1aaatCvAUfKttLearuWrP9MDH5MBPbIqV92AaeXatLxBI9gBaebbnrfifHhDYfgasaacH8akY=wiFfYdH8Gipec8Eeeu0xXdbba9frFj0=OqFfea0dXdd9vqai=hGuQ8kuc9pgc9s8qqaq=dirpe0xb9q8qiLsFr0=vr0=vr0dc8meaabaqaciaacaGaaeqabaqabeGadaaakeaadaWcaaqaaiabdAgaMnaaBaaaleaacqWGPbqAaeqaaOGaeiikaGIaemiEaG3aaSbaaSqaaiabdUgaRbqabaGccqGHRaWkcqWG0baDcqWGKbazdaWgaaWcbaGaem4AaSgabeaakiabcMcaPiabgkHiTiabdAgaMnaaBaaaleaacqWGPbqAaeqaaOGaeiikaGIaemiEaG3aaSbaaSqaaiabdUgaRbqabaGccqGGPaqkaeaacqWG0baDcqGHhis0cqWGMbGzdaWgaaWcbaGaemyAaKgabeaakiabcIcaOiabdIha4naaBaaaleaacqWGRbWAaeqaaOGaeiykaKYaaWbaaSqabeaacqWGubavaaGccqWGKbazdaWgaaWcbaGaem4AaSgabeaaaaGccqGHLjYSiiGacqWFXoqycqGGUaGlaaa@5569@

Since ∇*f*_*i*_(*x*_*k*_)^*T*^*d*_*k *_< 0, we deduce:

*f*_*i*_(*x*_*k *_+ *td*_*k*_) ≤ *f*_*i*_(*x*_*k*_) + *αt*∇*f*_*i*_(*x*_*k*_)^*T*^*d*_*k*_.

But *f*_*min*_(*x*_*k *_+ *td*_*k*_) ≤ *f*_*i*_(*x*_*k *_+ *td*_*k*_) and *f*_*min*_(*x*_*k*_) = *f*_*i*_(*x*_*k*_), so:

*f*_*min*_(*x*_*k *_+ *td*_*k*_) ≤ *f*_*min*_(*x*_*k*_) + *αt*∇*f*_*i*_(*x*_*k*_)^*T*^*d*_*k*_

for *t *small enough.

Therefore, choosing *t*_*k *_as in Steps 3.1–3.3, the condition (5) is satisfied.

This proves that, whenever *x*_*k *_is not critical, a point *x*_*k*+1 _satisfying (5) may be found, so the algorithm is well defined.

**Theorem 2 **If x_* _*is a limit point of a sequence generated by Algorithm ***A1 **then x_* _*is critical. Moreover, if *lim_*k*∈*K *_*x*_*k *_= x_* _*and the same i *= *ν*(*k*) ∈ *I*_*min*_(*x*_*k*_) *is chosen at Step 1 of the algorithm for infinitely many indices k *∈ *K*, *then i *∈ *I*_*min*_(x_*_) *and *∇*f*_*i*_(x_*_) = 0. *Finally*,

lim⁡k∈K‖∇fν(k)(xk)‖=0.
 MathType@MTEF@5@5@+=feaafiart1ev1aaatCvAUfKttLearuWrP9MDH5MBPbIqV92AaeXatLxBI9gBaebbnrfifHhDYfgasaacH8akY=wiFfYdH8Gipec8Eeeu0xXdbba9frFj0=OqFfea0dXdd9vqai=hGuQ8kuc9pgc9s8qqaq=dirpe0xb9q8qiLsFr0=vr0=vr0dc8meaabaqaciaacaGaaeqabaqabeGadaaakeaadaWfqaqaaiGbcYgaSjabcMgaPjabc2gaTbWcbaGaem4AaSMaeyicI4Saem4saSeabeaakmaafmaabaGaey4bIeTaemOzay2aaSbaaSqaaGGaciab=17aUjabcIcaOiabdUgaRjabcMcaPaqabaGccqGGOaakcqWG4baEdaWgaaWcbaGaem4AaSgabeaakiabcMcaPaGaayzcSlaawQa7aiabg2da9iabicdaWiabc6caUaaa@47AF@

*Proof*. Let x_* _∈ ℛ
 MathType@MTEF@5@5@+=feaafiart1ev1aaatCvAUfKttLearuWrP9MDH5MBPbIqV92AaeXatLxBI9gBamrtHrhAL1wy0L2yHvtyaeHbnfgDOvwBHrxAJfwnaebbnrfifHhDYfgasaacH8akY=wiFfYdH8Gipec8Eeeu0xXdbba9frFj0=OqFfea0dXdd9vqai=hGuQ8kuc9pgc9s8qqaq=dirpe0xb9q8qiLsFr0=vr0=vr0dc8meaabaqaciaacaGaaeqabaWaaeGaeaaakeaaimaacqWFBeIuaaa@377D@^*n *^be a limit point of the sequence generated by Algorithm **A1**. Let *K *= {*k*_0_, *k*_1_, *k*_2_, *k*_3_, ...} be an infinite sequence of integers such that:

1. There exists *i *∈ {1, ..., *m*} such that *i *= *ν*(*k*) for all *k *∈ *K*.

2. lim_*k*∈*K *_*x*_*k *_= x_*_.

The sequence *K *and the index *i *necessarily exist since {1, ..., *m*} is finite.

By the continuity of *f*_*i*_,

lim⁡k∈Kfi(xk)=fi(x∗).
 MathType@MTEF@5@5@+=feaafiart1ev1aaatCvAUfKttLearuWrP9MDH5MBPbIqV92AaeXatLxBI9gBaebbnrfifHhDYfgasaacH8akY=wiFfYdH8Gipec8Eeeu0xXdbba9frFj0=OqFfea0dXdd9vqai=hGuQ8kuc9pgc9s8qqaq=dirpe0xb9q8qiLsFr0=vr0=vr0dc8meaabaqaciaacaGaaeqabaqabeGadaaakeaadaWfqaqaaiGbcYgaSjabcMgaPjabc2gaTbWcbaGaem4AaSMaeyicI4Saem4saSeabeaakiabdAgaMnaaBaaaleaacqWGPbqAaeqaaOGaeiikaGIaemiEaG3aaSbaaSqaaiabdUgaRbqabaGccqGGPaqkcqGH9aqpcqWGMbGzdaWgaaWcbaGaemyAaKgabeaakiabcIcaOiabdIha4naaBaaaleaacqGHxiIkaeqaaOGaeiykaKIaeiOla4caaa@45D5@

Clearly, since *i *= *ν*(*k*), we have that

*f*_*i*_(*x*_*k*_) ≤ *f*_ℓ_(*x*_*k*_) for all ℓ ∈ {1, ..., *m*}.

for all *k *∈ *K*.

Taking limits on both sides of this inequality, we see that *f*_*i*_(x_*_) ≤ *f*_ℓ_(x_*_) for all ℓ ∈ {1, ..., *m*}. Thus,

*i *∈ *I*_*min *_(x_*_).

By the definition of Algorithm **A1**, since *k*_*j*+1 _≥ *k*_*j *_+ 1, we have:

fi(xkj+1)=fmin(xkj+1)≤fmin(xkj+1)≤fmin(xkj)+αtkj∇fi(xkj)Tdkj<fmin(xkj)=fi(xkj)
 MathType@MTEF@5@5@+=feaafiart1ev1aaatCvAUfKttLearuWrP9MDH5MBPbIqV92AaeXatLxBI9gBaebbnrfifHhDYfgasaacH8akY=wiFfYdH8Gipec8Eeeu0xXdbba9frFj0=OqFfea0dXdd9vqai=hGuQ8kuc9pgc9s8qqaq=dirpe0xb9q8qiLsFr0=vr0=vr0dc8meaabaqaciaacaGaaeqabaqabeGadaaakeaafaqaceWabaaabaGaemOzay2aaSbaaSqaaiabdMgaPbqabaGccqGGOaakcqWG4baEdaWgaaWcbaGaem4AaS2aaSbaaWqaaiabdQgaQjabgUcaRiabigdaXaqabaaaleqaaOGaeiykaKIaeyypa0JaemOzay2aaSbaaSqaaGqaciab=1gaTjab=LgaPjab=5gaUbqabaGccqGGOaakcqWG4baEdaWgaaWcbaGaem4AaS2aaSbaaWqaaiabdQgaQjabgUcaRiabigdaXaqabaaaleqaaOGaeiykaKIaeyizImQaemOzay2aaSbaaSqaaiab=1gaTjab=LgaPjab=5gaUbqabaGccqGGOaakcqWG4baEdaWgaaWcbaGaem4AaS2aaSbaaWqaaiabdQgaQjabgUcaRiabigdaXaqabaaaleqaaOGaeiykaKcabaGaeyizImQaemOzay2aaSbaaSqaaiab=1gaTjab=LgaPjab=5gaUbqabaGccqGGOaakcqWG4baEdaWgaaWcbaGaem4AaS2aaSbaaWqaaiabdQgaQbqabaaaleqaaOGaeiykaKIaey4kaSccciGae4xSdeMaemiDaq3aaSbaaSqaaiabdUgaRnaaBaaameaacqWGQbGAaeqaaaWcbeaakiabgEGirlabdAgaMnaaBaaaleaacqWGPbqAaeqaaOGaeiikaGIaemiEaG3aaSbaaSqaaiabdUgaRnaaBaaameaacqWGQbGAaeqaaaWcbeaakiabcMcaPmaaCaaaleqabaGaemivaqfaaOGaemizaq2aaSbaaSqaaiabdUgaRnaaBaaameaacqWGQbGAaeqaaaWcbeaaaOqaaiabgYda8iabdAgaMnaaBaaaleaacqWFTbqBcqWFPbqAcqWFUbGBaeqaaOGaeiikaGIaemiEaG3aaSbaaSqaaiabdUgaRnaaBaaameaacqWGQbGAaeqaaaWcbeaakiabcMcaPiabg2da9iabdAgaMnaaBaaaleaacqWGPbqAaeqaaOGaeiikaGIaemiEaG3aaSbaaSqaaiabdUgaRnaaBaaameaacqWGQbGAaeqaaaWcbeaakiabcMcaPaaaaaa@92CF@

for all *j *∈ N
 MathType@MTEF@5@5@+=feaafiart1ev1aaatCvAUfKttLearuWrP9MDH5MBPbIqV92AaeXatLxBI9gBamrtHrhAL1wy0L2yHvtyaeHbnfgDOvwBHrxAJfwnaebbnrfifHhDYfgasaacH8akY=wiFfYdH8Gipec8Eeeu0xXdbba9frFj0=OqFfea0dXdd9vqai=hGuQ8kuc9pgc9s8qqaq=dirpe0xb9q8qiLsFr0=vr0=vr0dc8meaabaqaciaacaGaaeqabaWaaeGaeaaakeaaimaacqWFneVtaaa@383B@.

By (5), (10) and (12), we obtain:

lim⁡j→∞tkj∇fi(xkj)Tdkj=0.
 MathType@MTEF@5@5@+=feaafiart1ev1aaatCvAUfKttLearuWrP9MDH5MBPbIqV92AaeXatLxBI9gBaebbnrfifHhDYfgasaacH8akY=wiFfYdH8Gipec8Eeeu0xXdbba9frFj0=OqFfea0dXdd9vqai=hGuQ8kuc9pgc9s8qqaq=dirpe0xb9q8qiLsFr0=vr0=vr0dc8meaabaqaciaacaGaaeqabaqabeGadaaakeaadaWfqaqaaiGbcYgaSjabcMgaPjabc2gaTbWcbaGaemOAaOMaeyOKH4QaeyOhIukabeaakiabdsha0naaBaaaleaacqWGRbWAdaWgaaadbaGaemOAaOgabeaaaSqabaGccqGHhis0cqWGMbGzdaWgaaWcbaGaemyAaKgabeaakiabcIcaOiabdIha4naaBaaaleaacqWGRbWAdaWgaaadbaGaemOAaOgabeaaaSqabaGccqGGPaqkdaahaaWcbeqaaiabdsfaubaakiabdsgaKnaaBaaaleaacqWGRbWAdaWgaaadbaGaemOAaOgabeaaaSqabaGccqGH9aqpcqaIWaamcqGGUaGlaaa@4DDF@

Therefore, by (2),

lim⁡j→∞tkj‖∇fi(xkj)‖‖dkj‖=0.
 MathType@MTEF@5@5@+=feaafiart1ev1aaatCvAUfKttLearuWrP9MDH5MBPbIqV92AaeXatLxBI9gBaebbnrfifHhDYfgasaacH8akY=wiFfYdH8Gipec8Eeeu0xXdbba9frFj0=OqFfea0dXdd9vqai=hGuQ8kuc9pgc9s8qqaq=dirpe0xb9q8qiLsFr0=vr0=vr0dc8meaabaqaciaacaGaaeqabaqabeGadaaakeaadaWfqaqaaiGbcYgaSjabcMgaPjabc2gaTbWcbaGaemOAaOMaeyOKH4QaeyOhIukabeaakiabdsha0naaBaaaleaacqWGRbWAdaWgaaadbaGaemOAaOgabeaaaSqabaGcdaqbdaqaaiabgEGirlabdAgaMnaaBaaaleaacqWGPbqAaeqaaOGaeiikaGIaemiEaG3aaSbaaSqaaiabdUgaRnaaBaaameaacqWGQbGAaeqaaaWcbeaakiabcMcaPaGaayzcSlaawQa7amaafmaabaGaemizaq2aaSbaaSqaaiabdUgaRnaaBaaameaacqWGQbGAaeqaaaWcbeaaaOGaayzcSlaawQa7aiabg2da9iabicdaWiabc6caUaaa@52C5@

If, for some subsequence *K*_1 _⊂ *K*, lim_*k*∈1 _∇*f*_*i*_(*x*_*k*_) = 0, we deduce that ∇*f*_*i*_(x_*_) = 0 and the thesis is proved. Therefore, we only need to analyze the possibility that ||∇*f*_*i*_(*x*_*k*_)|| is bounded away from zero for *k *∈ *K*. In this case, by (12),

lim⁡k∈Ktk‖dk‖=0.
 MathType@MTEF@5@5@+=feaafiart1ev1aaatCvAUfKttLearuWrP9MDH5MBPbIqV92AaeXatLxBI9gBaebbnrfifHhDYfgasaacH8akY=wiFfYdH8Gipec8Eeeu0xXdbba9frFj0=OqFfea0dXdd9vqai=hGuQ8kuc9pgc9s8qqaq=dirpe0xb9q8qiLsFr0=vr0=vr0dc8meaabaqaciaacaGaaeqabaqabeGadaaakeaadaWfqaqaaiGbcYgaSjabcMgaPjabc2gaTbWcbaGaem4AaSMaeyicI4Saem4saSeabeaakiabdsha0naaBaaaleaacqWGRbWAaeqaaOWaauWaaeaacqWGKbazdaWgaaWcbaGaem4AaSgabeaaaOGaayzcSlaawQa7aiabg2da9iabicdaWiabc6caUaaa@40FA@

If, for some subsequence, ||*d*_*k*_|| → 0, the condition (2) also implies that ∇*f*_*i*_(*x*_*k*_) → 0 and ∇*f*_*i*_(x_*_) = 0. Thus, we only need to consider the case in which lim_*k*∈*K *_*t*_*k *_= 0. Without loss of generality, we may assume that *t*_*k *_< 1 for all *k *∈ *K*. So, for all *k *∈ *K *there exists t¯k
 MathType@MTEF@5@5@+=feaafiart1ev1aaatCvAUfKttLearuWrP9MDH5MBPbIqV92AaeXatLxBI9gBaebbnrfifHhDYfgasaacH8akY=wiFfYdH8Gipec8Eeeu0xXdbba9frFj0=OqFfea0dXdd9vqai=hGuQ8kuc9pgc9s8qqaq=dirpe0xb9q8qiLsFr0=vr0=vr0dc8meaabaqaciaacaGaaeqabaqabeGadaaakeaacuWG0baDgaqeamaaBaaaleaacqWGRbWAaeqaaaaa@2FC0@ > 0 such that

fi(xk+t¯kdk)≥fmin(xk+t¯kdk)>fmin(xk)+αt¯k∇fi(xk)Tdk=fi(xk)+αt¯k∇fi(xk)Tdk.
 MathType@MTEF@5@5@+=feaafiart1ev1aaatCvAUfKttLearuWrP9MDH5MBPbIqV92AaeXatLxBI9gBaebbnrfifHhDYfgasaacH8akY=wiFfYdH8Gipec8Eeeu0xXdbba9frFj0=OqFfea0dXdd9vqai=hGuQ8kuc9pgc9s8qqaq=dirpe0xb9q8qiLsFr0=vr0=vr0dc8meaabaqaciaacaGaaeqabaqabeGadaaakeaacqWGMbGzdaWgaaWcbaGaemyAaKgabeaakiabcIcaOiabdIha4naaBaaaleaacqWGRbWAaeqaaOGaey4kaSIafmiDaqNbaebadaWgaaWcbaGaem4AaSgabeaakiabdsgaKnaaBaaaleaacqWGRbWAaeqaaOGaeiykaKIaeyyzImRaemOzay2aaSbaaSqaaiabd2gaTjabdMgaPjabd6gaUbqabaGccqGGOaakcqWG4baEdaWgaaWcbaGaem4AaSgabeaakiabgUcaRiqbdsha0zaaraWaaSbaaSqaaiabdUgaRbqabaGccqWGKbazdaWgaaWcbaGaem4AaSgabeaakiabcMcaPiabg6da+iabdAgaMnaaBaaaleaacqWGTbqBcqWGPbqAcqWGUbGBaeqaaOGaeiikaGIaemiEaG3aaSbaaSqaaiabdUgaRbqabaGccqGGPaqkcqGHRaWkiiGacqWFXoqycuWG0baDgaqeamaaBaaaleaacqWGRbWAaeqaaOGaey4bIeTaemOzay2aaSbaaSqaaiabdMgaPbqabaGccqGGOaakcqWG4baEdaWgaaWcbaGaem4AaSgabeaakiabcMcaPmaaCaaaleqabaGaemivaqfaaOGaemizaq2aaSbaaSqaaiabdUgaRbqabaGccqGH9aqpcqWGMbGzdaWgaaWcbaGaemyAaKgabeaakiabcIcaOiabdIha4naaBaaaleaacqWGRbWAaeqaaOGaeiykaKIaey4kaSIae8xSdeMafmiDaqNbaebadaWgaaWcbaGaem4AaSgabeaakiabgEGirlabdAgaMnaaBaaaleaacqWGPbqAaeqaaOGaeiikaGIaemiEaG3aaSbaaSqaaiabdUgaRbqabaGccqGGPaqkdaahaaWcbeqaaiabdsfaubaakiabdsgaKnaaBaaaleaacqWGRbWAaeqaaOGaeiOla4caaa@898C@

Moreover, by (13),

lim⁡k∈Kt¯k‖dk‖=0.
 MathType@MTEF@5@5@+=feaafiart1ev1aaatCvAUfKttLearuWrP9MDH5MBPbIqV92AaeXatLxBI9gBaebbnrfifHhDYfgasaacH8akY=wiFfYdH8Gipec8Eeeu0xXdbba9frFj0=OqFfea0dXdd9vqai=hGuQ8kuc9pgc9s8qqaq=dirpe0xb9q8qiLsFr0=vr0=vr0dc8meaabaqaciaacaGaaeqabaqabeGadaaakeaadaWfqaqaaiGbcYgaSjabcMgaPjabc2gaTbWcbaGaem4AaSMaeyicI4Saem4saSeabeaakiqbdsha0zaaraWaaSbaaSqaaiabdUgaRbqabaGcdaqbdaqaaiabdsgaKnaaBaaaleaacqWGRbWAaeqaaaGccaGLjWUaayPcSdGaeyypa0JaeGimaaJaeiOla4caaa@4112@

Define *s*_*k *_= t¯k
 MathType@MTEF@5@5@+=feaafiart1ev1aaatCvAUfKttLearuWrP9MDH5MBPbIqV92AaeXatLxBI9gBaebbnrfifHhDYfgasaacH8akY=wiFfYdH8Gipec8Eeeu0xXdbba9frFj0=OqFfea0dXdd9vqai=hGuQ8kuc9pgc9s8qqaq=dirpe0xb9q8qiLsFr0=vr0=vr0dc8meaabaqaciaacaGaaeqabaqabeGadaaakeaacuWG0baDgaqeamaaBaaaleaacqWGRbWAaeqaaaaa@2FC0@*d*_*k *_for all *k *∈ *K*. Then, by (15),

lim⁡k∈K‖sk‖=0.
 MathType@MTEF@5@5@+=feaafiart1ev1aaatCvAUfKttLearuWrP9MDH5MBPbIqV92AaeXatLxBI9gBaebbnrfifHhDYfgasaacH8akY=wiFfYdH8Gipec8Eeeu0xXdbba9frFj0=OqFfea0dXdd9vqai=hGuQ8kuc9pgc9s8qqaq=dirpe0xb9q8qiLsFr0=vr0=vr0dc8meaabaqaciaacaGaaeqabaqabeGadaaakeaadaWfqaqaaiGbcYgaSjabcMgaPjabc2gaTbWcbaGaem4AaSMaeyicI4Saem4saSeabeaakmaafmaabaGaem4Cam3aaSbaaSqaaiabdUgaRbqabaaakiaawMa7caGLkWoacqGH9aqpcqaIWaamcqGGUaGlaaa@3E12@

By (14) and the Mean Value Theorem, for all *k *∈ *K *there exists *ξ*_*k *_∈ [0, 1] such that

∇*f*_*i*_(*x*_*k *_+ *ξ*_*k*_*s*_*k*_)^*T*^*s*_*k *_= *f*_*i*_(*x*_*k *_+ *s*_*k*_) - *f*_*i*_(*x*_*k*_) > *α*∇*f*_*i*_(*x*_*k*_)^*T*^*s*_*k*_.

Moreover, by (2),

∇fi(xk)Tsk‖sk‖≤−θ‖∇fi(xk)‖
 MathType@MTEF@5@5@+=feaafiart1ev1aaatCvAUfKttLearuWrP9MDH5MBPbIqV92AaeXatLxBI9gBaebbnrfifHhDYfgasaacH8akY=wiFfYdH8Gipec8Eeeu0xXdbba9frFj0=OqFfea0dXdd9vqai=hGuQ8kuc9pgc9s8qqaq=dirpe0xb9q8qiLsFr0=vr0=vr0dc8meaabaqaciaacaGaaeqabaqabeGadaaakeaadaWcaaqaaiabgEGirlabdAgaMnaaBaaaleaacqWGPbqAaeqaaOGaeiikaGIaemiEaG3aaSbaaSqaaiabdUgaRbqabaGccqGGPaqkdaahaaWcbeqaaiabdsfaubaakiabdohaZnaaBaaaleaacqWGRbWAaeqaaaGcbaWaauWaaeaacqWGZbWCdaWgaaWcbaGaem4AaSgabeaaaOGaayzcSlaawQa7aaaacqGHKjYOcqGHsisliiGacqWF4oqCdaqbdaqaaiabgEGirlabdAgaMnaaBaaaleaacqWGPbqAaeqaaOGaeiikaGIaemiEaG3aaSbaaSqaaiabdUgaRbqabaGccqGGPaqkaiaawMa7caGLkWoaaaa@5131@

for all *k *∈ *K*.

Let *K*_1 _⊂ *K*, *s *∈ ℛ
 MathType@MTEF@5@5@+=feaafiart1ev1aaatCvAUfKttLearuWrP9MDH5MBPbIqV92AaeXatLxBI9gBamrtHrhAL1wy0L2yHvtyaeHbnfgDOvwBHrxAJfwnaebbnrfifHhDYfgasaacH8akY=wiFfYdH8Gipec8Eeeu0xXdbba9frFj0=OqFfea0dXdd9vqai=hGuQ8kuc9pgc9s8qqaq=dirpe0xb9q8qiLsFr0=vr0=vr0dc8meaabaqaciaacaGaaeqabaWaaeGaeaaakeaaimaacqWFBeIuaaa@377D@^*n *^be such that lim⁡k∈K1sk/‖sk‖=s
 MathType@MTEF@5@5@+=feaafiart1ev1aaatCvAUfeBSjuyZL2yd9gzLbvyNv2CaerbwvMCKfMBHbqedmvETj2BSbqee0evGueE0jxyaibaieIgFLIOYR2NHOxjYhrPYhrPYpI8F4rqqrFfpeea0xe9Lq=Jc9vqaqpepm0xbbG8FasPYRqj0=yi0lXdbba9pGe9qqFf0dXdHuk9fr=xfr=xfrpiWZqaaeaabiGaaiaacaqabeaabeqacmaaaOqaaiGacYgacaGGPbGaaiyBamaaBaaaleaacaWGRbGaeyicI4Saam4samaaBaaameaacaaIXaaabeaaaSqabaGccaWGZbWaaSbaaSqaaiaadUgaaeqaaOGaai4lamaafmaabaGaam4CamaaBaaaleaacaWGRbaabeaaaOGaayzcSlaawQa7aiabg2da9iaadohaaaa@46CE@.

By (16), dividing both sides of the inequality (17) by ||*s*_*k*_||, and taking limits for *k *∈ *K*_1_, we obtain:

∇*f*_*i*_(x_*_)^*T*^*s *≥ *α*∇*f*_*i*_(x_*_)^*T*^*s*.

Since *α *< 1 and ∇*f*_*i*_(*x*_*k*_)^*T*^*d*_*k *_< 0 for all *k*, this implies that ∇*f*_*i*_(x_*_)^*T*^*s *= 0. Thus, taking limits in (18), we obtain that ∇*f*_*i*_(x_*_) = 0. Therefore, by (11), x_* _is critical.

Finally, let us prove (9). If (9) is not true, there exists *j *and an infinite set of indices *k *∈ *K *such that *j *= *ν*(*k*) and ||∇ *f*_*j*_(*x*_*k*_)|| is bounded away from zero. This implies that *j *∈ *I*_*min*_(x_*_) and ||∇*f*_*j*_(x_*_)|| ≠ 0, contradicting the first part of the proof.    □

## Availability and requirements

The program for performing protein structural alignments with these methods is freely available, with source codes, at:



An *online server *for pairwise comparison is also available. The methods are implemented in such a way that performing pairwise, single-protein-to-database or all-on-all database comparisons is straightforward.

## Authors' contributions

LM proposed that the alignment problem could be treated by LOVO theory. LM, RA and JMM designed the algorithms. LM and JMM have written the program and LM has performed the numerical experiments. RA and JMM developed theoretical aspects. LM and JMM have written the paper. All authors have approved the final manuscript.
